# High Resolution Manofluorographic Study in Patients With Multiple System Atrophy: Possible Early Detection of Upper Esophageal Sphincter and Proximal Esophageal Abnormality

**DOI:** 10.3389/fmed.2018.00286

**Published:** 2018-10-05

**Authors:** Rumi Ueha, Takao Goto, Taku Sato, Nogah Nativ-Zeltzer, Shih Chieh Shen, Takaharu Nito, Peter C. Belafsky, Tatsuya Yamasoba

**Affiliations:** ^1^Department of Otolaryngology, The University of Tokyo, Tokyo, Japan; ^2^Department of Otolaryngology, The University of California, Davis, Davis, CA, United States

**Keywords:** multiple system atrophy, high resolution manofluorography, swallowing, upper esophageal sphincter, proximal esophageal abnormality, pharyngoesophageal abnormality, abnormal deglutitive proximal esophageal contraction

## Abstract

**Introduction:** Multiple system atrophy (MSA) has detrimental effects on swallowing function. The swallowing function of patients with MSA has not been systematically characterized and the underlying pathophysiological mechanisms of dysphagia remain poorly understood.

**Objectives:** To investigate the characteristics of swallow function in MSA using high-resolution manofluorography (HRMF).

**Methods:** We conducted a retrospective review of twenty-five MSA patients who underwent HRMF from 2016 to 2017. HRMF was utilized on patients with only oral diet (Functional Oral Intake Scale (FOIS) >3). Pharyngoesophageal and proximal esophageal pressure profiles were evaluated and compared to established normative data. The frequency and characteristics of upper esophageal sphincter (UES) and proximal esophageal abnormalities during rest and swallow were calculated.

**Results:** The ages of patient cohort in our study ranged from 48–81 years (median 65 years) with male predominance (68%). We observed a distinct abnormal deglutitive proximal esophageal contraction (ADPEC) in 14 (56% of patients), which appears to reflect a discoordinated response of the striated muscle esophagus. Deficient UES relaxation duration, impaired UES relaxation, hypertensive resting UES pressure and hypotensive resting UES pressure were detected in 8 patients (32%), 3 patients (12%), 1 patient (4%), and 11 patients (44%) respectively.

**Conclusions:** In patients with MSA, abnormal UES resting pressure is common. A discoordinated proximal esophageal pressure response was identified and may be a pathognomonic manometry finding for MSA. These findings may serve as indications of early stage swallowing dysfunction in patients with MSA.

## Introduction

Multiple system atrophy (MSA) is a neurodegenerative disorder that causes systemic degeneration of the cerebellar, extrapyramidal and autonomic nervous systems in various combinations (1) and is generally classified as parkinsonism predominance (MSA-P) and/or cerebellar ataxia (MSA-C) (2).

Deterioration of swallowing function is one of the manifestations of MSA that dramatically affects quality of life and prognosis of patients (3) and dysphagia- related complications such as respiratory infections (mostly aspiration pneumonia) and malnutrition are most the common causes of death (4, 5). Delayed oral and pharyngeal phases of swallowing, in combination with impaired airway protection and esophageal sphincter contraction disturbances, may lead to acute aspiration and pneumonia (6). Impaired relaxation of the upper esophageal sphincter (UES) has been reported regardless of MSA phenotype (7). It is hypothesized that neurodegenerative changes in the brain stem and vagus nerve might contribute to a dysfunctional cricopharyngeous muscle, resulting in impairment of UES opening and bolus residue in the pyriform sinuses (3, 6, 7). Despite the prevalence of swallow dysfunction in persons with MSA, swallowing function in MSA has not been investigated systematically, and the underlying pathophysiological mechanisms of dysphagia, especially the incipient swallowing changes in MSA, remain poorly understood. Early detection of dysphagia, prevention of aspiration pneumonia and possible intervention can increase quality of life and survival time of MSA patients.

Technological advancements and utilizations of high-resolution manometry (HRM) have led to a better understanding of pharyngeal and esophageal disorders. HRM has been utilized to characterize swallowing dysfunction in patients with neuromuscular disease, such as Parkinson's disease but not in MSA. Moreover, high-resolution manofluorography (HRMF), an armamentarium combining high-resolution manometric and videofluoroscopic swallowing examinations, improves the accuracy of diagnosis of esophageal disorders and facilitates dysphagia management (8, 9). The purpose of this investigation was to evaluate the characteristic pharyngoesophageal and cervical esophageal findings of HRMF in patients with MSA.

## Methods

### Ethics

This study was approved by the Human Ethics Committee of the University of Tokyo (No. 2487). Written informed consent was obtained from every patient and patients' anonymities were preserved.

### Patients

Twenty-five MSA patients whose diagnosis was made at the Department of Neurology in our institute between the years 2016 to 2017 were included. Patients' diagnoses were confirmed according to the Consensus Statement of diagnostic criteria and excluded from Gilman's criteria (2).

### Methodology

We conducted a retrospective review of patients with MSA who underwent HRMF at the University of Tokyo Hospital. Clinical and demographic profiles were analyzed, including age, gender, type, and severity stage of MSA (10), presence of vocal fold immobility, Functional Oral Intake Scale (FOIS) (11), Penetration Aspiration Scale (PAS) score (12) in thin liquid intake (10 mPa•s) and thickened liquid intake (200 mPa•s) and HRMF parameters.

MSA severity was designated from Stage 1 to 5 as previously reported (10). FOIS scores (11), and PAS scores (12) were assigned as Level 1–7 (normal level: 7) and Score 1–8 (most severe score: 8) separately (Table 1).

**Table 1:** Details of severity stages of Multiple System Atrophy (10), a Functional Oral Intake Scale (11), and a Penetration Aspiration Scale (12).

**Severity stages of multiple system atrophy**	
Stage 1	Able to walk without support
Stage 2	Aid-required walking, with use of a walking aid or a companion's arm for support, but not at all times
Stage 3	Aid-required walking, with use, at all times, of a walking aid or a companion's arm for support
Stage 4	Wheelchair-bound state (wheelchair use at all times)
Stage 5	Bedridden state (complete loss of ability for independent activity)
**Functional oral intake scale**	
Level 1	No oral intake
Level 2	Tube dependent with minimal/inconsistent oral intake
Level 3	Tube supplements with consistent oral intake
Level 4	Total oral intake of a single consistency
Level 5	Total oral intake of multiple consistencies requiring special preparation
Level 6	Total oral intake with no special preparation, but must avoid specific foods or liquid items
Level 7	Total oral intake with no restrictions
**Penetration-aspiration scale**	
Score 1	Material does not enter airway
Score 2	Material enters the airway, remains above the vocal folds, and is ejected from the airway
Score 3	Material enters the airway, remains above the vocal folds, and is not ejected from the airway
Score 4	Material enters the airway, contacts the vocal folds, and is ejected from the airway
Score 5	Material enters the airway, contacts the vocal folds, and is not ejected from the airway
Score 6	Material enters the airway, passes below the vocal folds, and is ejected into the larynx or out of the airway
Score 7	Material enters the airway, passes below the vocal folds, and is not ejected from the trachea despite effort
Score 8	Material enters the airway, passes below the vocal folds, and no effort is made to eject

### High resolution manofluorographic study and measures

HRMF studies were performed on all MSA patients who were on an oral diet (FOIS >3) in the upright position. The manometric catheter was lubricated with 4% viscous lidocaine and inserted transnasally. The videofluoroscopic swallow study (VFSS) was conducted in the lateral view simultaneously. The protocol consisted of a 5-min baseline recording, followed by 3 dry swallows and 3 wet swallows of 5 cc thickened contrast agent (iohexol: Omnipaque®, Daiichi-Sankyo, Tokyo, Japan). A solid-state high-resolution manometer (Starlet, Star Medical, Tokyo, Japan) was used for all data acquisition. The manometric catheter (Unisensor, Portsmouth, NH, USA) has an outer diameter of 4 mm and 20 circumferential pressure sensors spaced 1 cm apart. The data acquisition frequency was 40 Hz for each sensor. The system is calibrated to record pressures between −50 and 300 mmHg. Video was recorded at a rate of 30 frames/s and analyzed by Star.exeve 8.1 (Star Medical, Tokyo, Japan). Pharyngeal and proximal esophageal measures were obtained on all patients.

**Figure 1:**
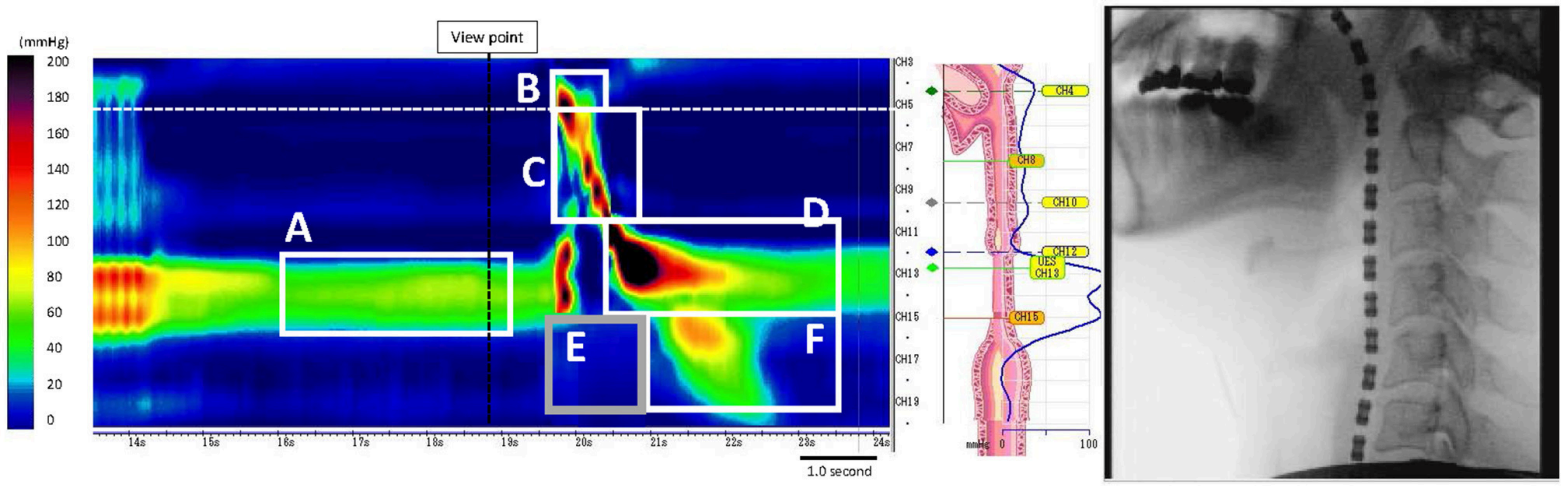
Measurement of UES pressure and each integral (in a healthy subject). **(A)** Resting UES pressure, **(B)** VCI, Velopharyngeal closure integral, **(C)** MHCI, Meso-hypopharynx contractile integral, **(D)** PDUCI, Post-deglutitive UES contractile integral, **(E)** PECIDS, Proximal esophageal contractile integral during swallowing, **(F)** PPECI, Post-deglutitive proximal esophageal contractile integral.

The following parameters were obtained from the manometric output: maximum swallowing pressures (velopharynx, meso-hypopharynx, post-deglutitive UES pressure), integrals [velopharyngeal closure integral, meso-hypopharynx contractile integral, post-deglutitive UES contractile integral, proximal esophageal contractile integral during UES relaxation (early response), proximal esophageal contractile integral], UES relaxation duration, UES relaxation pressure (nadir) and UES resting pressure (Figure 1).

Abnormal HRMF metrics in the UES and proximal esophagus were identified using the following criteria: impaired UES relaxation pressure/duration (relaxation pressure >12 mmHg/<0.45 s), hypertensive resting UES pressure (>104 mmHg), and hypotensive resting UES pressure (<34 mmHg) (13, 14). Proximal esophageal contraction integral above 0 mmHg during UES opening (an early response occurring concurrently with UES opening) was also defined as abnormal, a phenomenon observed in this study among MSA patients. The frequency of abnormal hypertensive and discoordinated abnormal proximal esophageal contraction during swallowing (ADPEC), deficient UES relaxation duration, UES abnormalities, and other sub-types of UES abnormal presentations on manometry were calculated. Finally, subjects were divided into two groups based on presence or absence of ADPEC and intergroup comparison of HRMF parameters were made and correlations between ADPEC and clinical features between groups were evaluated.

### Statistical analysis

Statistical comparisons between groups were performed by using Mann–Whitney *U*-tests implemented in GraphPad Prism (version 6.0; GraphPad Software, Inc., La Jolla, CA, USA). Demographic variables (type of MSA, vocal cord immobility) were compared by Fisher's exact test between patients with or without ADPEC. *P* < 0.05 was considered statistically significant.

## Results

### Demographic data

Data from the charts of 25 patients with MSA were abstracted. The age of the cohort (Table 2) ranged from 48 to 81 years (median 65 years). Sixty-eight percent (17/25) was male. Seven out of 25 patients were diagnosed with MSA-P (Parkinsonian features predominant type MSA), and the remaining 18 patients were diagnosed with MSA-C (cerebellar symptoms predominant type MSA). Regarding MSA severity, 7 patients were stage 1 or 2, 10 patients were stage 3 and 8 patients were designated stage 4. Bilateral vocal fold immobility was detected in 8 patients (32%). Total oral nutritional dependence was noted in all patients.13 patients (52%) had no restrictions (FOIS 7), 8 patients (32%) had minimal dietary restrictions (FOIS 6), and 4 patients (16%) had moderate diet modification (FOIS 5). For thin liquids, 15 patients (60%) had PAS 1-3, 5 patients (20%) had a PAS of 4-6, and 5 patients (20%) had a PAS of 7 or 8. For thickened liquids, 20 patients (80%) had a normal PAS (PAS 1), 3 patients (12%) had a PAS of 2, and 2 patients (8%) had a PAS of 6.

**Table 2:** Demographic data and high-resolution manofluorography (HRMF) findings of upper esophageal sphincter (UES) and proximal esophageal metrics in multiple system atrophy (MSA) patients.

**No**.	**Age**	**Sex**	**Type**	**Severity stage**	**Vocal cord immobility**	**FOIS**	**PAS**		**APECDS**	**UES opening**	**UES relaxation**	**Resting UES pressure**
							**Liquid**	**Thickened**				
1	65	M	MSA-C	2	No	7	1	1	+	Normal	Normal	Hypotensive
2	62	M	MSA-C	1	No	7	1	1	+	Deficient	Normal	Normal
3	57	M	MSA-C	3	No	7	1	1	−	Normal	Normal	Normal
4	53	M	MSA-C	2	No	7	1	1	−	Normal	Impaired	Normal
5	55	M	MSA-C	2	No	7	1	1	−	Normal	Normal	Normal
6	53	M	MSA-C	3	No	7	1	1	+	Normal	Normal	Normal
7	47	F	MSA-C	3	Bilateral	7	1	1	+	Normal	Normal	Hypotensive
8	77	M	MSA-C	3	No	7	1	1	+	Normal	Normal	Normal
9	70	M	MSA-P	2	Bilateral	7	1	1	−	Normal	Normal	Normal
10	67	M	MSA-P	2	No	7	1	1	+	Deficient	Normal	Hypotensive
11	64	M	MSA-P	3	No	7	1	1	−	Normal	Normal	Hypotensive
12	66	M	MSA-P	3	No	7	1	1	+	Normal	Normal	Hypotensive
13	65	F	MSA-P	4	Bilateral	7	1	1	+	Deficient	Normal	Hypotensive
14	70	F	MSA-C	4	Bilateral	6	2	1	−	Normal	Normal	Hypotensive
15	68	M	MSA-C	3	Bilateral	6	2	1	+	Normal	Normal	Hypotensive
16	64	M	MSA-C	2	No	6	4	1	+	Normal	Normal	Normal
17	79	F	MSA-P	3	No	6	4	1	+	Normal	Normal	Normal
18	70	F	MSA-C	4	Bilateral	6	6	1	−	Normal	Normal	Normal
19	68	M	MSA-C	4	Bilateral	5	6	2	+	Deficient	Normal	Hypotensive
20	56	F	MSA-C	4	No	5	6	2	+	Deficient	Normal	Normal
21	81	F	MSA-P	3	Bilateral	6	7	1	−	Normal	Normal	Normal
22	55	M	MSA-C	4	No	5	7	6	−	Normal	Normal	Normal
23	60	F	MSA-C	4	No	6	8	1	−	Deficient	Normal	Hypotensive
24	68	M	MSA-C	4	No	6	8	2	−	Deficient	Impaired	Hypotensive
25	56	M	MSA-C	3	No	5	8	6	+	Deficient	Impaired	Hypertensive

*MSA-P, Parkinsonian features predominant type MSA; MSA-C, Cerebellar symptoms predominant type MSA; FOIS, Functional Oral Intake Scale; PAS, Penetration Aspiration Scale; ADPEC, Abnorma deglutitive proximal esophageal contraction*.

### HRMF pharyngeal and proximal esophageal metrics in MSA patients

Mean, standard deviation and the measured ranges for each pharyngeal and proximal esophageal metric from patients with MSA included in this study are shown in Table 3. The maximum swallowing pressures and integrals of each segment, UES relaxation duration, resting UES pressure and UES relaxation pressure (nadir) were included. Pharyngeal contraction and UES status were verified for each study by VFSS in order to minimize measurement errors.

**Table 3:** Statistical summary of HRMF from patients with MSA.

	**Mean ±*SD* (5–95th percentile)**
**Maximum pressure (mmHg)**
Velopharynx	222 ± 70 (193–251)
Meso-hypopharynx	324 ± 115 (277–372)
Post-deglutitive UES	417 ± 115 (369–464)
**Integral (mmHg/s/cm)**	
VCI	121 ± 60 (97–146)
MHCI	300 ± 118 (251–348)
PDUCI	636 ± 402 (470–802)
PECIDS	53 ± 77 (21–85)
PPECI	83 ± 105 (40–127)
Resting UES pressure (mmHg)	44 ± 35 (30–59)
UES opening duration (ms)	473 ± 112 (427–519)
UES relaxation pressure-nadir (mmHg)	−0.8 ± 18.2 (−8.3–6.7)

*Values are mean ± SD (5–95th percentile). UES, Upper esophageal sphincter; VCI, Velopharyngeal closure integral; MHCI, Meso-hypopharynx contractile integral; PDUCI, Post-deglutitive UES contractile integral; PECIDS, Proximal esophageal contractile integral during swallowing; PPECI, Post-deglutitive proximal esophageal contractile integral*.

### Abnormal HRMF findings of UES and proximal esophageal metrics

**Figure 2:**
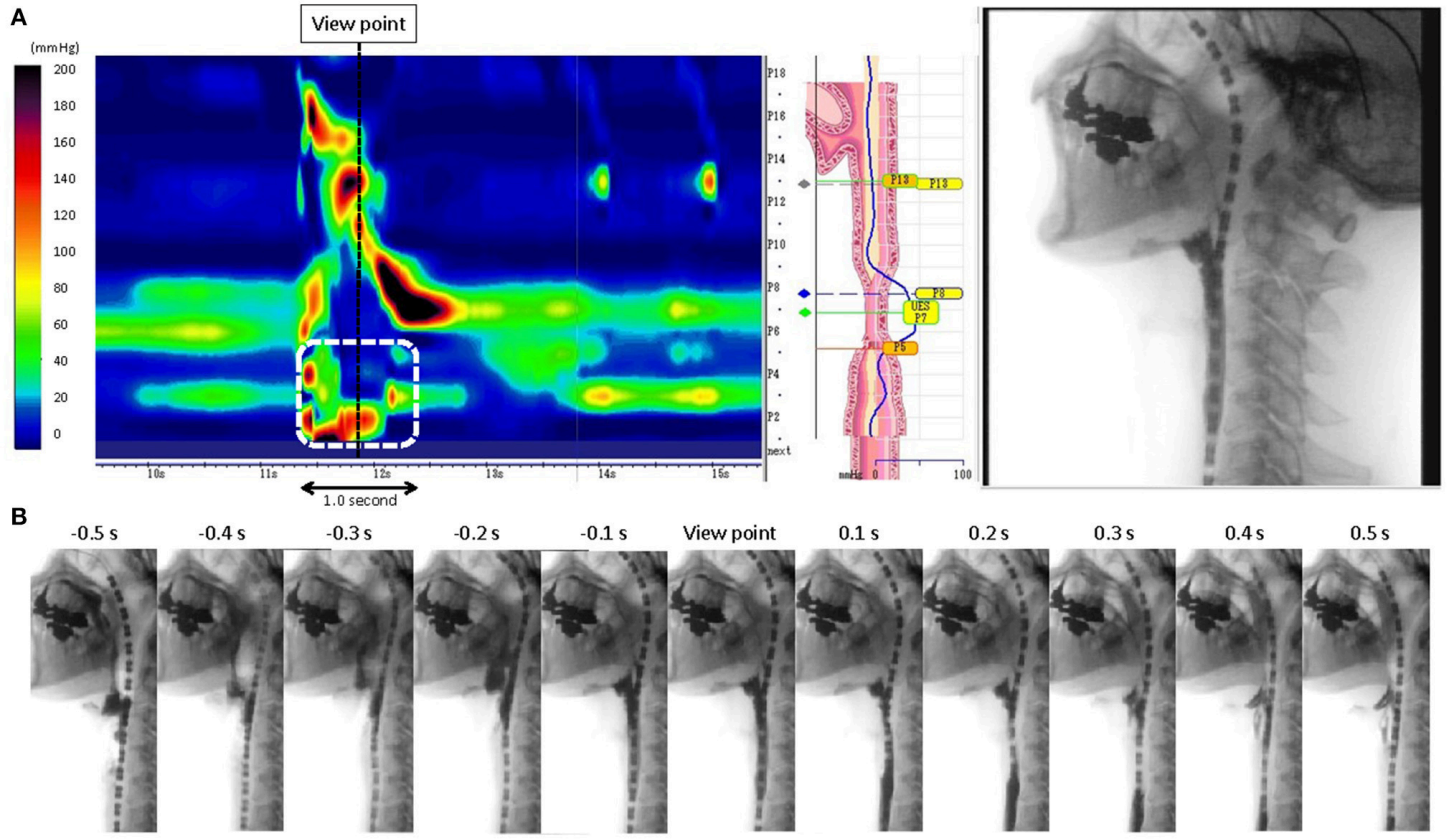
**(A)** Representative pattern of abnormal hypertensive and discoordinated proximal esophageal contraction during swallowing (ADPEC: The area surrounded by a white broken line). A black broken line shows the view point of the static fluoroscopic image. **(B)** 1.0 s time series images of fluoroscopic examination.

HRMF measurements of UES and proximal esophageal metrics on pressure topography are illustrated in Figure 1. HRMF revealed a pattern of abnormal hypertensive and discoordinated proximal esophageal contraction during swallowing (ADPEC) (Figure 2) in 14 patients (56%). Deficient UES relaxation duration, impaired UES relaxation, hypertensive resting UES pressure and hypotensive resting UES pressure were detected in 8 patients (32%), 3 patients (12%), 1 patient (4%), and 11 patients (44%), respectively.

### Comparison of clinical features and HRMF values between groups with and without abnormal hypertensive and discoordinated proximal esophageal contraction during swallowing

There were no statistically significant differences in any of the HRMF values between groups with or without ADPEC. Similarly, no significant associations were found between the presence of ADPEC and types of MSA (OR 0.94; 95% CI 0.16–5.46; *p* = 1.00) or presence of vocal fold immobility (OR 0.70; 95% CI 0.13–3.79; *p* = 0.69). There was no significant difference in severity stages of MSA between groups with and without ADPEC (Table 4).

**Table 4:** Comparison of HRMF values between groups with/without abnormal discoordinated deglutitive proximal esophageal contraction (ADPEC) and associations of ADPEC and type of MSA, vocal cord immobility.

	**Non-ADPEC**	**ADPEC**	***P*-value**
**Maximum pressure (mmHg)**
Velopharynx	201 ± 68 (156–247)	230 ± 70 (190–271)	0.62
Meso-hypopharynx	334 ± 127 (249–420)	327 ± 122 (257–398)	0.78
Post-deglutitive UES	407 ± 97 (342–472)	434 ± 136 (355–514)	0.9
**Integral (mmHg/s/cm)**			
VCI	117 ± 51 (82–151)	122 ± 65 (85–160)	0.78
MHCI	290 ± 146 (192–379)	299 ± 77 (255–344)	0.66
PDUCI	631 ± 530 (275–986)	614 ± 313 (433–795)	0.7
PPECI	99 ± 145 (14–196)	72 ± 62 (36–108)	0.68
Resting UES pressure (mmHg)	37 ± 2 (21–53)	50 ± 41 (26–74)	0.74
UES opening duration (ms)	510 ± 86 (453–568)	443 ± 124 (372–515)	0.14
UES relaxation pressure-nadir (mmHg)	−1.8 ± 12.1 (-9.9–6.2)	−0.0 ± 22.2 (−12.9–12.8)	0.66
MSA-C/MSA-P	8/3	10/4	1.00
VCIm/Non-VCIm	4/7	4/10	0.69
Severity stage	2.9 ± 0.86	3.2 ± 0.87	0.38

*Values are mean ± SD (5–95th percentile). UES, Upper esophageal sphincter; VCI, Velopharyngeal closure integral; MHCI, Meso-hypopharynx contractile integral; PDUCI, Post-deglutitive UES contractile integral; PECIDS, Proximal esophageal contractile integral during swallowing; PPECI, Post-deglutitive proximal esophageal contractile integral; MSA-P, Parkinsonian features predominant type MSA; MSA-C, Cerebellar symptoms predominant type MSA; VCIm, vocal cord immobility*.

## Discussion

Swallowing dysfunction is common in patients with MSA. Appropriate evaluations for dysphagia may prevent or delay complications such as aspiration pneumonia and sudden death. In this study, abnormal UES pressures and a pattern of discoordinated proximal esophageal contraction pressure during swallowing and resting were identified in MSA patients with oral nutritional status (FOIS score >3). These findings did not correlate with the severity stage, types of MSA, or concurrent vocal fold immobility.

MSA is a progressive neurodegenerative disease and can affect swallowing function variably with disease progression. The disordered oral stage of swallowing manifests in delayed bolus transport to the pharynx and worsens progressively due to bradykinesia and disrupted tongue coordination (15). Diminished oropharyngeal and hypopharyngeal manometric swallowing pressures of various degrees have been reported (7). The current investigation supports these data and suggests that pharyngeal pressures on HRMF are variable. In addition, another study showed 23.1% of MSA patients had incomplete relaxation of the UES (7). Deficient UES relaxation duration and impaired UES relaxation were also found in certain MSA patients in this study, however, the pathophysiology of how MSA affected the UES remains incompletely understood.

MSA can also cause esophageal stage impairments such as dysmotility (16). It has been proposed that preganglionic cholinergic dysfunction and specifically loss of the dorsal vagal nucleus innervating the digestive system lead to food stagnation within the esophagus (17) and occur more prevalently in MSA patients with dysphagia (16). Thus, evaluation of the esophageal phase is also indicated.

Complex sequences of swallowing are controlled and regulated by the medullary swallowing center, which is also known as the central pattern generators (CPGs) (18). The finding of ADPEC in MSA patients in this study may be an example of abnormal or discoordinated motility stemming from central nervous system dysfunction. Additional investigations are necessary to obtain a better understanding of the mechanism of ADPEC in patients with MSA and other neurodegenerative disease.

Vocal fold immobility is another frequent complication of MSA (3). Swallowing dysfunction and vocal fold immobility are related, as both the thyropharyngeal and cricopharyngeal muscles are innervated by the vagus nerve (3, 7). Few studies have evaluated the relationship between vocal fold immobility and dysphagia in MSA. We previously reported that vocal fold abductor paralysis tended to precede dysphagia in MSA-C patients (19) and the MSA-C patients with severe vocal fold abductor paralysis also suffered from severe dysphagia (3). Impaired UES opening and bolus stasis at the pyriform sinuses have been reported as associated with vocal fold abductor paralysis in MSA (7). In this study, however, no significant association was found between ADPEC and vocal fold immobility. This could result from the restriction of patients with only mild or moderate severity MSA in our cohort. An association between ADPEC and vocal fold immobility may be more common in patients with high severity MSA.

HRMF can improve understanding and advance our capabilities of evaluating the pharyngo-esophageal region. In this study, concurrent UES and proximal esophageal abnormalities, possibly due to neuromuscular dysfunction, were frequently noted even in mild MSA patients, and ADPEC identified on HRMF may be suggestive of MSA. Our findings suggest that HRMF may assist with the earlier detection of deglutitive abnormalities before the symptom of dysphagia develops. However, it is not fully clear whether the ADPEC is an early indication of pathophysiologic and neurodegenerative changes in MSA patients, an indicator of MSA severity, or possibly a compensatory proximal esophageal contraction in response to ongoing pharyngo-esophageal dysmotility. Longitudinal observations are required to elucidate the clinical significance of ADPEC in patients with MSA.

MSA affects the autonomic nervous system and swallowing function progressively. Pulmonary complications, malnourishments, or even mortality is related to clinical deterioration. The data suggest that HRMF may be beneficial for the early detection of swallowing dysfunction in MSA patients. Further investigation is required to confirm the accuracy of HRMF in the early detection of MSA and assess the effect of early detection on wellness and quality of life.

## Conclusions

UES pressure anomalies and a discoordinated proximal esophageal pressure response are common manometric findings in persons with MSA. Further investigation is required to investigate the mechanisms underlying this pressure pattern and validate the application of HRMF as a diagnostic tool for MSA.

## Author contributions

RU developed the concept, designed and performed the experiments, and analyzed the data. TN, PB, and TY developed the concept, designed the experiments. TG, TS, NN-Z, and SS performed the experiments, and analyzed the data. All authors contributed to interpretation of the data and writing of the manuscript.

### Conflict of interest statement

The authors declare that the research was conducted in the absence of any commercial or financial relationships that could be construed as a potential conflict of interest.
